# Sorghum *Brown Midrib19* (*Bmr19*) Gene Links Lignin Biosynthesis to Folate Metabolism

**DOI:** 10.3390/genes12050660

**Published:** 2021-04-28

**Authors:** Adedayo O. Adeyanju, Scott E. Sattler, Patrick J. Rich, Luis A. Rivera-Burgos, Xiaochen Xu, Gebisa Ejeta

**Affiliations:** 1 Department of Agronomy, Purdue University, 915 W. State St., West Lafayette, IN 47907-2054, USA; aadeday@purdue.edu (A.O.A.); pjrich@purdue.edu (P.J.R.); lariver2@ncsu.edu (L.A.R.-B.); xu449@purdue.edu (X.X.); 2 Wheat, Sorghum & Forage Research Unit, USDA-ARS, East Campus, University of Nebraska-Lincoln, 251 Filley Hall East, Lincoln, NE 68583-0937, USA; scott.sattler@usda.gov

**Keywords:** sorghum, brown midrib mutants, *bmr19*, lignin, lignocellulosic feedstock, folylpolyglutamate synthase

## Abstract

Genetic analysis of brown midrib sorghum (*Sorghum bicolor*) mutant lines assembled in our program has previously shown that the mutations fall into four allelic groups, *bmr2*, *bmr6*, *bmr12* or *bmr19*. Causal genes for allelic groups *bmr2*, *bmr6* and *bmr12*, have since been identified. In this report, we provide evidence for the nature of the *bmr19* mutation. This was accomplished by introgressing each of the four *bmr* alleles into nine different genetic backgrounds. Polymorphisms from four resequenced bulks of sorghum introgression lines containing either mutation, relative to those of a resequenced bulk of the nine normal midrib recurrent parent lines, were used to locate their respective causal mutations. The analysis confirmed the previously reported causal mutations for *bmr2* and *bmr6* but failed in the case of *bmr12*-bulk due to a mixture of mutant alleles at the locus among members of that mutant bulk. In the *bmr19*-bulk, a common G → A mutation was found among all members in *Sobic.001G535500*. This gene encodes a putative folylpolyglutamate synthase with high homology to maize *Bm4.* The brown midrib phenotype co-segregated with this point mutation in two separate F_2_ populations. Furthermore, an additional variant allele at this locus obtained from a TILLING population also showed a brown midrib phenotype, confirming this locus as *Bmr19*.

## 1. Introduction

### 1.1. Biological Role of Lignin

Lignin is a complex heteropolymer of phenylpropanoid-derived monolignols providing support and water-conducting ability to the cell wall by cross-linking with its polysaccharide components cellulose (polymers of glucose) and hemicellulose (heteropolymers of other monosaccharides including arabinose, glucose, mannose and xylose). Together these cell wall components form the lignocellulosic matrix that constitutes the greatest part of dry plant matter. The lignocellulosic matrix varies greatly between plant species and organs of individual plants [1]. The cross linking provided by lignin in grass secondary cell walls allows herbaceous panicoids to reach up to several meters in height. Leaf lignin content is highest in sheaths and midribs of the leaf blades making these more rigid to support a plant architecture that maximizes photosynthetic capacity [2,3]. The lignin constituent is particularly diverse, involving several possible linkages among the basic monolignol subunits [1].

### 1.2. Significance of Lignin in Biofuel Production

Among the many ethnobotanical uses of lignocellulosic biomass is feedstock in the production of ethanol as an environmentally friendly alternative to liquid fossil fuels. Carbon emissions of lignocellulosic ethanol is arguably half of gasoline when considered over the entire production and use chain [4]. Both technical and transport logistic concerns limit its economic viability, however. Chief among these concerns is the processing needed to expose the cellulose and hemicellulose to saccharification, thereby releasing their sugars for fermentation to ethanol. Lignin not only shields the polysaccharides from hydrolysis, it also binds the added hydrolytic enzymes, which impedes saccharification [5]. Feedstock from tissues of highly lignified cell walls require more input for the same ethanol yield than less fortified tissues.

### 1.3. Biosynthesis of Lignin, Composition of Monolignols and Their Polymerization

Monolignol subunits differ in the number of methyoxy groups on their aromatic rings, syringyl (S) having two, guaiacyl (G) with one and, peculiar to grasses, *p*-hydroxyphenyl (H) subunits with none [5]. The cinnamyl alcohol dehydrogenase (CAD2) catalyzes the reduction of hydroxycinnamyl aldehydes from their corresponding alcohols (monolignols) [6]. The aldehyde precursors are synthesized in the cytosol from phenylalanine or tyrosine through a series of steps, one of which is catalyzed by 4-coumarate: coenzyme A ligase (4CL) [7]. Two *O*-methyl transferases, caffeoyl coenzyme A 3-*O*-methyl transferase (CCoAOMT) and caffeic acid-*O*-methyl transferase (COMT) catalyze the addition of methoxy groups at 3 and 5 positions of phenol ring using *S*-adenosyl methionine (SAM) as the methyl donor [8]. Polymerization of monolignols occurs within the cell wall through radical-coupled oxidation of the monolignols [9]. In grasses, phenolic compounds *p*-coumaric acid and ferulic acid derived from this pathway are also incorporated into cell walls and can be linked to lignin polymers [2].

### 1.4. Brown Midrib Mutations

Mutations affecting lignin biosynthesis in maize (*Zea mays*), sorghum (*Sorghum bicolor*) and pearl millet (*Pennisetum glaucum*) phenotypes are identified by the reddish-brown leaf midribs and stalk fibers [10]. Recently, this phenotype was also reported in rice (*Oryza sativa*) carrying a mutation in a *CAD2* gene [11]. The cause of this color change from the normal green or white is not understood, but this change may be due to incorporation of hydroxycinnamaldehydes into the lignin polymer [12].

### 1.5. Allelic Groups among the bmr Mutants of Sorghum

An original set of 19 sorghum brown midrib (*bmr*) mutants were first described in our sorghum research program from two lines chemically mutagenized in efforts to improve forage quality [13]. Since then, we have added a few more lines through mutagenesis and selections from natural variants in different source populations. A total of 28 brown midrib sorghum variants were later subjected to an allelism test determining that the collection of mutants we had assembled fall into four allelic groups (*bmr2*, *bmr6*, *bmr12* and *bmr19*) with altered lignin content [14]. An additional four allelic groups (*bmr29* through *bmr32*) have also been isolated subsequently [15].

The *Bmr2* gene of sorghum was identified as *Sobic.004G062500*, encoding a 4CL and two distinct missense mutations in *Bmr2* were described [7]. Leaf midrib lignin content of *bmr2* mutants is 16% less than wildtype [14]. Lignin of *bmr2* mutants have about half the concentration of G-lignin as wildtype with a slight, though less consistent, reduction in S-lignin [14]. The glucose yield after saccharification with cellulose hydrolyzing enzymes was increased in *bmr2* mutant biomass over wildtype by 17% [14].

The *Bmr6* gene of sorghum was identified as *Sobic.004G071000*, encoding CAD2. Its homolog in maize is *Bm1* [16]. At least eight distinct *bmr6* alleles have been described [3,6,17,18,19]. Leaf midrib lignin content of *bmr6* mutants is 19% less than wildtype with the G-lignin subunits disproportionately reduced relative to S-lignin [14]. Saccharification yields an average of 16% more glucose than wildtype in *bmr6* mutants [14]. This translates to an increased ethanol conversion efficiency of 44% [20].

The *Bmr12* gene of sorghum was identified as *Sobic.007G047300*, encoding a COMT [21] and its homolog in maize is *Bm3* [22]. Nine distinct mutant *bmr12* alleles have been identified [14,21,23,24,25]. Leaf midrib lignin content of *bmr12* mutants averages about 13% less than wildtype [14]. The cell wall of the *bmr12* mutants contains fewer S-lignin subunits and less esterified *p*-coumaric acid (preferentially esterified to S-lignin) relative to wildtype [14,21]. This translates to an increased ethanol conversion efficiency of 46% [20]. These mutants also have significantly less tricin than wildtype, a feature indicative of the hypothesized role of COMT in tricin biosynthesis [26]. Saccharification is improved by as much as 24% over wildtype in *bmr12* mutants [14].

The *Bmr19* gene of sorghum was not previously identified and only one allele was identified in allelism tests [14]. Leaf midrib lignin content of *bmr19* mutants is only slightly (−3%) less than wildtype [14]. The *bmr19* mutant midrib has a similar lignin composition to *bmr2* mutants, having a more modest reduction in G-lignin and a slight reduction in S-lignin relative to wildtype [14]. Saccharification was not improved in *bmr19* stover and may have even yielded less glucose than wildtype [14]. In this report, we identify the gene that encodes *Bmr19* as a putative folylpolyglutamate synthase (FPGS) gene, which has high amino acid sequence similarity to maize *Bm4* (Phytozome v.12). This evidence is based on whole-genome sequenced bulks from five different genetic backgrounds into which the four allelic groups were introgressed and their respective unconverted wild type parents (Table 1), as well as an additional *bmr19* allele at this locus from an EMS mutagenized TILLING population with the brown midrib phenotype.

## 2. Materials and Methods

### 2.1. Plant Material

A group of introgression lines in which mutations in each of the original sorghum *Bmr* genes (*bmr2-ref*, *bmr2-5*, *bmr6-ref*, *bmr6-3*, *bmr12-ref*, *bmr12-7*, *bmr12-18* and *bmr19-ref*) were backcrossed into nine different genetic backgrounds. The various brown midrib mutations were introduced in the original crosses using a genic male sterile (*ms3ms3*) version of each mutant donor as the female, except in a few crosses involving male sterile (*ms3ms3*) Tx623 and P9401 where the brown midrib line was used as the male (Table 1). From the F_2_ of each cross, selection was for the brown midrib phenotype and against male sterility. The donors of the *bmr2* mutations mainly carried the *bmr2-ref* allele, except in one lineage in which *bmr2-5* was used. The donors of *bmr6* lineages included either of two alleles, *bmr6-ref* or *bmr6-3*. The donors of *bmr12* lineages included either of three alleles, *bmr12-ref*, *bmr12-7* or *bmr12-18*. The *bmr19* donors all carried the original mutant allele (*bmr19-ref*). The male parents were chosen based on desirable agronomic characters and were pure lines with normal (non-brown) midrib phenotypes. These were used as recurrent parents during the backcrossing process.

### 2.2. DNA Extraction and Construction of the Bulked Pools

Two leaf discs of 0.6 cm diameter were punched from one plant representing each of the lineages, confirming the leaf midrib phenotype (brown or normal). Two leaf discs of nine plants (one from each lineage) were ground together according to the target *Bmr* gene in Table 1 with a mortar and pestle (16–18 discs) in liquid nitrogen. Five bulks were thus homogenized: the *bmr2* bulk with plants with the *bmr2*/*bmr2* genotype, the *bmr6* bulk sharing the genotype *bmr6*/*bmr6*, the *bmr12* bulk with *bmr12*/*bmr12*, the *bmr19* bulk with *bmr19*/*bmr19* and the normal bulk sharing wildtype alleles at all four *Bmr* loci (Table 1). DNA was isolated in 4 × 100 mg aliquots from each of the five homogenates in separate extractions using the Qiagen DNeasy Plant Mini Kit (Qiagen, Germantown, MD, USA). The smaller aliquots avoided overloading the capacity of the extraction kit units while providing enough DNA for sequencing. Equimolar concentrations of DNA from each of the four aliquots representing each bulk were combined and purified with the DNA Clean-Up & Concentration Kit (ZYMO Research, Irvine, CA, USA) before submitting for sequencing at the Purdue Genomics Core Facility, West Lafayette, Indiana.

### 2.3. Construction of Libraries and Illumina Sequencing

Libraries were prepared using TruSeq DNA PCR-Free LT Library Preparation Kit-Set B, FC-121-3002 (Illumina, San Diego, CA, USA). Two micrograms of DNA from each sample was sheared using a Covaris S2 ultrasonicator (Covaris, Woburn, MA, USA), end repaired and adapter ligated. Each sample was ligated to differently indexed adapters to allow them all to be run in the sample lane. Size selection of libraries was performed using polyethylene glycol cuts with the aid of magnetic binding beads as described in the kit protocol. This results in a target insert size of 500–600 bp among library molecules that cluster. The quality of the resulting DNA libraries was assessed on an Agilent Technologies 2100 Bioanalyzer using a High Sensitivity chip. Final libraries were titred for clustering using a KAPA Library Quantification Kit Illumina^®^ Platforms (KAPA Cat # KR045), pooled and clustered in 3.5 lanes of Illumina Hiseq 2500 High Output v3 chemistry (Illumina Inc., San Diego, CA, USA) to generate 100-base pair-end reads.

### 2.4. Construction of Reference-Based Assemblies

The statistics of generated sequencing reads was estimated using Samtools [27]. The Mutmap pipeline (http://genome-e.ibrc.or.jp/home/bioinformaticsteam/mutmap, accessed on 12 October 2019, developed by Iwate Biotechnology Research Center, Kitakami, Japan) was used for calculating the single nucleotide polymorphism (SNP)-index. Briefly, the cleaned reads of one the normal midrib parents which had been resequenced (Sudan Zera-Zera) were first aligned to the sorghum reference genome of BTx623 (PhytozomeV10: Sbicolor_313_v3.1.1) using the Burrows–Wheeler Aligner [28]. Coval 1.4 was used for post-processing and filtering of the alignment files [29]. The variants called were then used to develop a reference-based assembly of Sudan Zera-Zera by substituting the bases with confidence variants calls in the genome. The reads from the each of the *bmr* mutant bulks and the normal bulk were aligned separately and variants were called for both bulks against the developed reference assembly.

Calculation of SNP-index: a SNP-index for each SNP position was calculated for each bulk as per Abe et al. [30] using the formula:

SNP-index (at a position) = count of alternate base/count of reads aligned.

The SNPs in the candidate region close to the causal mutation are expected to have a higher SNP index (SNP-index ≈ 1); in contrast, those in unlinked regions show a SNP index of *c.* 0.5. A peak in the SNP index indicates the approximate genomic interval harboring the causal mutation. The positions with SNP-index < 0.3 and read depth < 0.7 in the bulks was filtered out for ΔSNP-index calculation. ΔSNP-index was calculated by subtracting SNP-index of each *bmr* bulk from SNP-index of the normal bulk. The ΔSNP-index is ≈1 if the bulked DNA comprises only *bmr* genome, ΔSNP-index is ≈ −1 if it is the normal parent genome only and ΔSNP-index = 0 if both bulks have the same SNP indices at the genomic regions. The possible effects of the SNPs (synonymous, missense and frameshift mutations) between the *bmr19* and normal bulks at candidate genomic regions were inferred using the predictCoding tool in the GenomicFeatures [31] and VariantAnnotation [32] open source software in R. The selected variants were compared to the variant data of 71 sorghum lines [33,34] available on Phytozome V 12.1. Our expectation was that our candidate causal SNP would not be present in the variant data since none of the deep sequenced lines have a brown midrib phenotype. Using this approach, we were able to eliminate several variants in the candidate region until we found the causal variant for each mutation. Individual sequence reads containing the causal variants were also viewed with IGV software (v.2.8.10) [35] where closely linked SNPs were expected to show 100% mutant and 0% normal-type reads.

### 2.5. Indel Markers of the Bmr12 Gene

Variation among members of the *bmr12* and normal bulks was examined by PCR followed by gel electrophoresis of the amplicons using primers that amplified two functionally inert indels within *Sobic.007G047300* [18,36]. The largest indel, present in the first intron is an *Olo24* retrotransposon present in the reference genome but absent in both of the lines (P954104 and P954114) mutagenized by Porter et al. [13] and therefore, also absent in the original 19 brown midrib mutant lines. This region was amplified with the primer pair (5′ → 3′) GACCGGACAGTGACTTCAGAG (forward) and GGACTGTTACTGCTGCCATGGC (reverse). Presence and absence of this element were distinguished by amplicons differing by 348 bp. The other polymorphism was in a SSR (TATC)_n_, also in the first intron, amplified by the primers TCCGAAGTGCTCAAGCCTAT (forward) and CAGTCGTGGAGGATCCACTT (reverse). The original brown midrib mutant lines vary from the reference at this position by two fewer repeat units (8 bp).

### 2.6. Validation of Identified bmr19 Mutant SNP

One genic SNP identified for the *bmr19* mutation was validated in nine parents used in the normal bulk and the nine *bmr19* lineages used to constitute the *bmr19* bulk (Table 1). The SNP was also validated in two F_2_ populations, (bmr19 × IS4225)F_2_ (*n* = 285) and (BTx623 × bmr19)F_2_ (*n* = 149) segregating for the brown midrib phenotype. For this purpose, the tetra-primer ARMS-PCR technique [37] was used to design allele specific primers that converted the SNP to a size polymorphism that could be detected by gel electrophoresis. This marker was also used on the original brown midrib lines of our collection (bmr2, bmr3, bmr4, bmr6, bmr7, bmr12, bmr14, bmr15, bmr18, bmr19, bmr20, bmr22, bmr23, bmr24, bmr25, bmr26, bmr27 and bmr28) to check for co-segregation of the SNP with the *bmr19*-specific brown-midrib phenotype.

### 2.7. Alternate bmr19 Alleles

Because only one mutant allele, *bmr19-ref*, in this group was available in our stocks, we took advantage of the sequenced M4 lines of a TILLING population resulting from ethyl methanesulfonate (EMS) mutagenesis of BTx623. According to the Functional Gene Discovery Platform for Sorghum created at Purdue University (https://www.purdue.edu/sorghumgenomics/, accessed on 12 October 2019), six EMS lines carry point mutations in coding regions of *Sobic.001G535500*. Four of these were obtained from the U.S. National Plant Germplasm System (NPGS), accessions PI 677943 (0623), PI 677955 (0662), PI 678119 (2354) and PI 678132 (2465). These were grown in pots in the greenhouse along with bmr19 and BTx623 as mutant and wildtype checks, respectively. By six weeks after sowing, midrib phenotypes were visually scored.

The only one of these EMS lines showing a brown midrib phenotype, 2354, carrying an allele that we named *bmr19-2*, was crossed onto an emasculated bmr19 plant and the resulting seed was sown in greenhouse pots. The putative F_1_ plants were confirmed as non-selfs by checking their DNA with the tetra-primers [37] used to track segregation of the *bmr19-ref* and *Bmr19* alleles in the F_2_ populations described in the validation section. True F_1_s give two allele specific amplicons (162 + 124 bp) by PCR, along with the control PCR product (233 bp), just like heterozygotes in the screened F_2_s. Progeny of any possible self-pollinated plants, therefore, would give two bands when their PCR products are separated on a gel: control (233 bp) and the *bmr19-ref* allele “A” at this position (162 bp), while the progeny of a cross gives three bands: control (233 bp), the *bmr19-ref* allele (162 bp) and the one for “G” at this position (124 bp). Because of the proximity of the *bmr19-2* and *bmr19-ref* point mutations (Table 2), the control amplicon spanned the location of both point mutations as does the 124 bp product unique to F_1_ plants. Sequencing of this latter PCR product was used to confirm the presence of the *bmr19-2* allele in the F_1_ plants. At six weeks, the midrib phenotype of these plants was visually scored.

### 2.8. Biomass Composition Analysis

Two sets of plants were used to compare effects of *bmr* mutations on lignin amount in dried biomass after removing mature grain (stover). The first set, grown in the greenhouse in the off-season included all lines of the BTx623 background [BTx623, (bmr2ms3 × Tx623B)BC_3_F_5_, (bmr6ms3 × Tx623B)BC_3_F_5_, (BTx623ms3 × bmr18)BC_2_F_4_, (BTx623ms3 × bmr19)BC_2_F_4_ and BTx623 EMS line 2354]. The second set included only those lines contrasting for variations in *Bmr19* [BTx623, (BTx623ms3 × bmr19)BC_2_F_4_ and BTx623 EMS line 2354]. This set was grown in the field during the regular season in West Lafayette. Above-ground plant parts were harvested at grain maturity, the panicles removed, stalks split length-wise and vegetative tissues (leaves and stems) chopped into 2” pieces and dried in a convection oven at 50⁰C for two weeks. Fiber analysis was performed on ground stover from each set to determine cell wall components using a detergent digestion protocol as described by Vogel et al. [38]. Neutral detergent fiber, acid detergent fiber and acid detergent lignin concentrations were estimated using the ANKOM 200 fiber analyzer (ANKOM Tech Co., Macedon, NY, USA) [38]. Relative percentage of individual cell wall components (cellulose, hemicellulose and lignin) were calculated using component concentrations extracted on a dry weight basis [39]. Stover from four biological replicates was analyzed in duplicate (technical reps) for the first set and five biological replicates in duplicate for the second set. Each biological replicate consisted of one plant. Stover from *bmr19* and wildtype plants of the second set were treated for thioacidolysis followed by gas chromatography-mass spectrometry (GC-MS) to determine relative lignin subunit composition (*p*-hydroxyphenyl, guaiacyl and syringyl lignin). Samples were prepared and analyzed as described by Palmer et al. [40]. Analysis was performed in duplicate on five biological replicates per line. SAS version 9.4 (SAS Institute, Cary, NC, USA) was used for statistical analysis of compositional data. The linear mixed model analysis of variance (ANOVA) with the Tukey–Kramer multiple comparison adjustment procedure was performed to compare lignin properties between genotypes.

## 3. Results

### 3.1. Confirmation of the bmr2-ref Allele in the bmr2 Bulk

**Figure 1:**
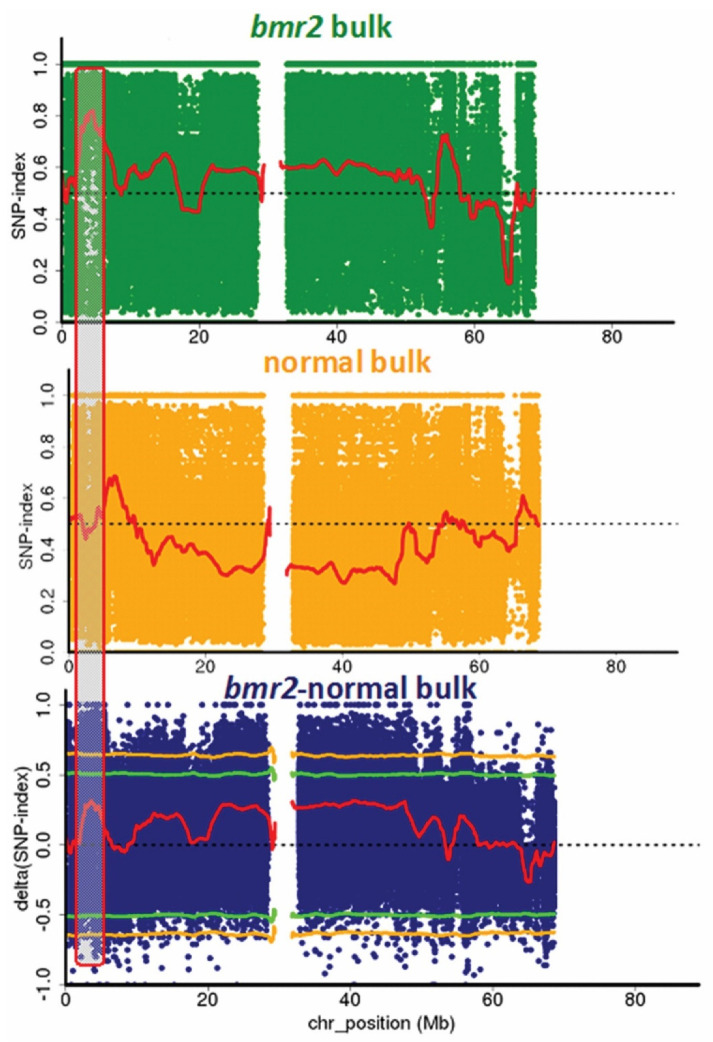
Single nucleotide polymorphism (SNP) analysis to identify *bmr2* mutation. SNP index plots of *bmr2* bulk (**top**), normal bulk (**middle**) and Δ(SNP-index) plot (**bottom**) of sorghum Chromosome 4 with statistical confidence intervals under the null hypothesis of no QTLs (green, *p* < 0.05; yellow, *p* < 0.01). Grey highlighted area shows the QTL near the beginning of Chromosome 4 where the significant difference between the bulks occurs.

Mutmap applied to the *bmr2* bulk revealed a SNP index peak in the genomic interval from 3 Mb to 6 Mb on Chromosome 4. Analysis of SNPs in the candidate region revealed the same SNP in *Sobic.004G062500*, encoding 4CL, identified by Saballos et al. [7] differentiating *bmr2* from the normal bulk. Plots of the SNP indices of the bulks and ΔSNP-index between them are shown in Figure 1. The consensus assemblies of the two bulks show a SNP at position Chr04:5,041,758 (Phytozome *Sorghum bicolor* v.3.1.1) with G → A on the forward strand predicted to cause the missense mutation G111D in the 4CL protein, the *bmr2-ref* allele [7]. Most reads in the *bmr2* bulk showed this allele (A) at this position while all those of the normal bulk had the wildtype allele (G) here. Donor lines of the brown midrib phenotype carried the *bmr2-ref* allele (Table 1), but one donor in this group was bmr5, a previous undefined mutant, which contains a C → T mutation at Chr04:5,042,645 resulting in the substitution H251Y called *bmr2-5* [14].

### 3.2. Confirmation of the bmr6-ref Allele in the bmr6 Bulk

**Figure 2:**
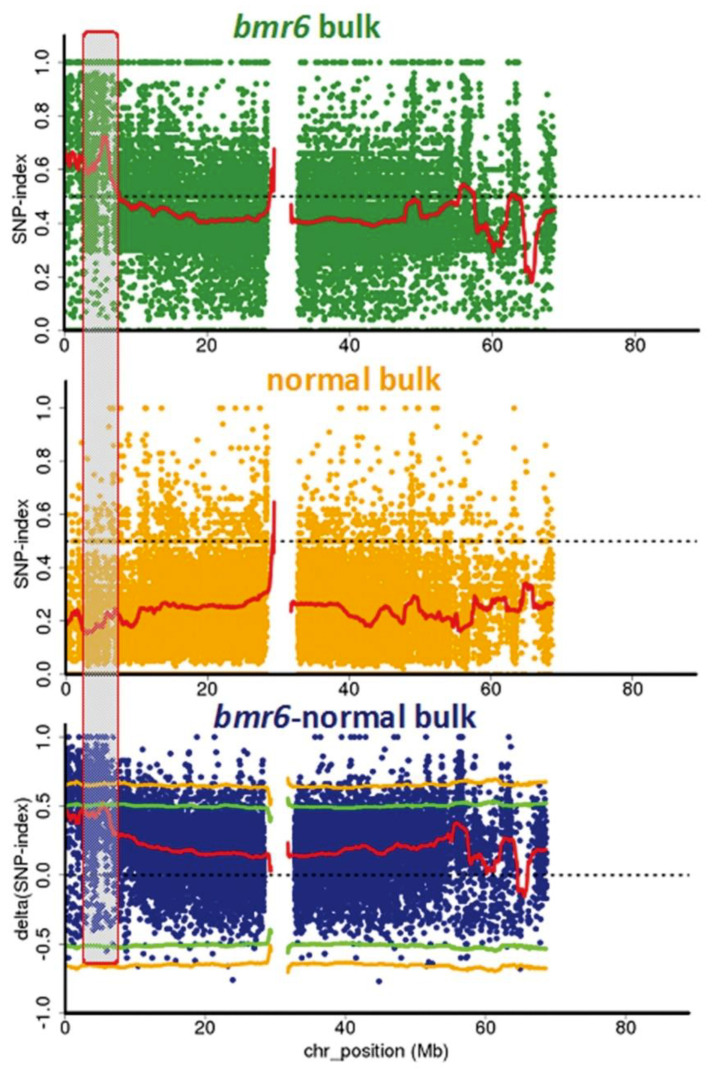
Single nucleotide polymorphism (SNP) analysis to identify *bmr6* mutation. SNP index plots of *bmr6* bulk (**top**), normal bulk (**middle**) and Δ(SNP-index) plot (**bottom**) of sorghum Chromosome 4 with statistical confidence intervals under the null hypothesis of no QTLs (green, *p* < 0.05; yellow, *p* < 0.01). Grey highlighted area shows the QTL near the beginning of Chromosome 4 where the significant difference between the bulks occurs.

The aligned consensus assemblies of the *bmr6* and normal bulks showed a SNP index peak in the genomic interval from 4Mb to 6.6 Mb on Chromosome 4. The analysis of SNPs in this candidate region revealed a candidate SNP corresponding to *Sobic.004G071000*, encoding CAD2. The presumed causal SNP at this locus between the bulks was at position Chr04:5,731,386 (Phytozome *Sorghum bicolor* v.3.1.1) with a C → T on the reverse strand, the same mutation defined as the *bmr6-ref* allele, resulting in a predicted nonsense mutation Q132stop [6]. The SNP indices plots for Chromosome 4 comparing the *bmr6* and normal bulks are shown in Figure 2. The highest peak of the ΔSNP-index plot between the *bmr6* and normal bulks defining this candidate region approached the *p* < 0.05 threshold, but like the *bmr2* comparison, fell short of this significance level due to the presence in the *bmr6* bulk of a second mutant allele, *bmr6-3* contributed by brown midrib mutant line bmr3 in the pedigrees of two members of the bulk (Table 1). This allele, G → A on the reverse strand at position Chr04:5,730,502 predicted to cause the missense mutation G129S [6], was present at a low frequency among the aligned reads in the *bmr6* bulk. Its frequency was low enough to prevent miscall of the consensus assembly comparison variation between the *bmr6* and normal bulks in the analysis.

### 3.3. Comparison of the bmr12 and Normal Bulks

The analysis failed to pick a consistent difference between the *bmr12* and normal bulks at the *Bmr12* gene, *Sobic.007G047300*, encoding for COMT [21]. Therefore, no candidate genomic region was defined for this comparison of bulks at this locus. This was likely due to a mixture of mutant alleles in the *bmr12* bulk and for variation among the members of the normal bulk for the indels in this gene described by Gorthy et al. [18]. We examined members of both the *bmr12* and normal bulks by primers that amplified the regions containing these indels [36]. Examination of the aligned reads at this locus shows a high frequency of both the *bmr12-18* (G → A on the reverse strand at position Chr07:4,724,050, at a frequency of 67%) and *bmr12-ref* (C → T on the reverse strand at position Chr07:4,723,997, at a frequency of 7%) defined by Bout and Vermerris [21] among the pooled reads of the *bmr12* bulk. A few reads (frequency = 17%) in this bulk also showed the *bmr12-7* allele [14], a C → T on the reverse strand at position Chr07:4,722,193.

### 3.4. Identification of the Bmr19 Gene

**Figure 3:**
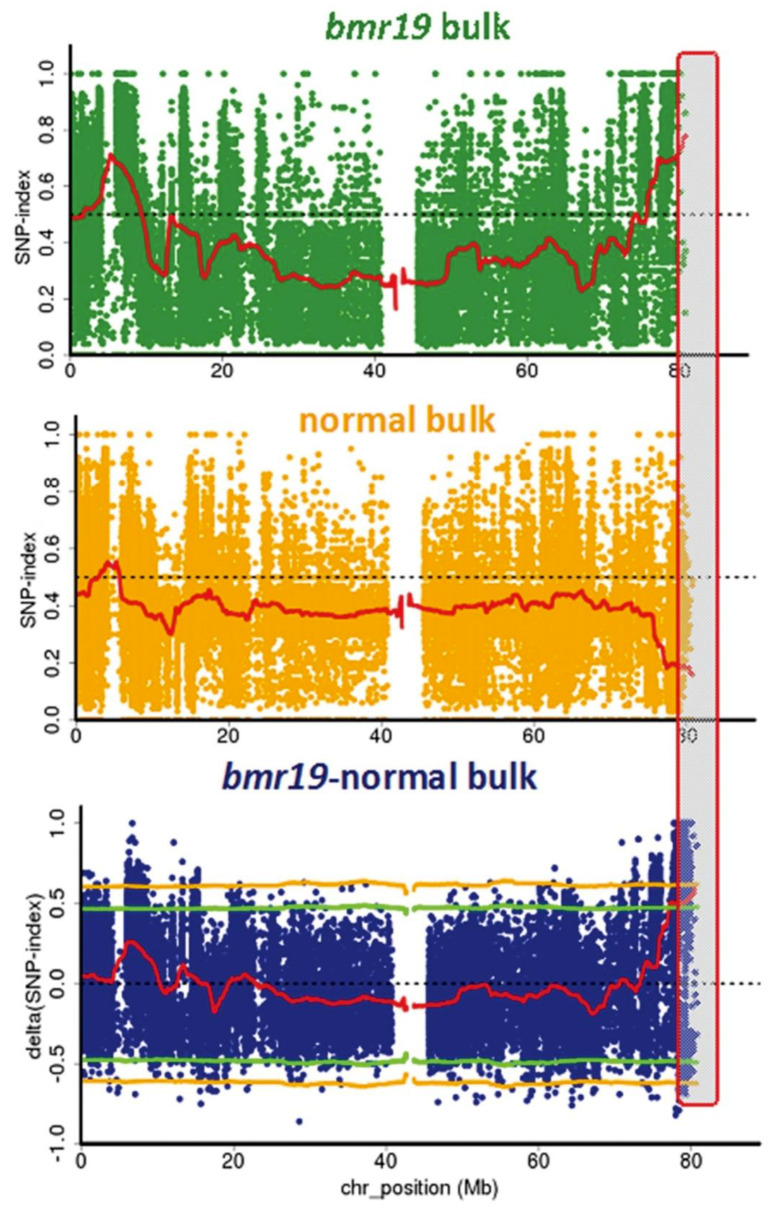
Single nucleotide polymorphism (SNP) analysis to identify *bmr19* mutation. SNP index plots of *bmr19* bulk (**top**), normal bulk (**middle**) and Δ(SNP-index) plot (**bottom**) of sorghum Chromosome 1 with statistical confidence intervals under the null hypothesis of no QTLs (green, *p* < 0.05; yellow, *p* < 0.01). Grey highlighted area shows the QTL at the end of Chromosome 1 where the significant difference between the bulks occurs.

The same analysis performed on the other brown midrib bulks indicated a SNP-index peak in the genomic interval from 70 Mb to 80 Mb on at the end of sorghum Chromosome 1 when the *bmr19* and normal bulks were compared (Figure 3). Analysis of SNPs in this candidate region revealed a candidate SNP G → A mutation on the forward strand at position Chr01:79,894,470 (Phytozome *Sorghum bicolor* v.3.1.1) in the predicted gene *Sobic.001G535500*. This gene encodes a putative folylpolyglutamate synthase (FPGS) with high homology (95.4% identical + positively scoring residues in alignment according to Phytozome v.12) to maize *Bm4*, *GRMZM2G393334* [41]. The maize *bm4* mutant has a brown midrib phenotype and, like *bmr19*, shows only slight changes in lignin amount and composition [41,42]. The mutation (*bmr19-ref*) defined by this SNP occurs in the seventh exon of the gene model (Phytozome *Sorghum bicolor* v3.1.1) and is predicted to cause the missense mutation G240E. The mutation occurs within the annotated Mur-ligase domain in a part associated with binding the glutamic acid. This mutation has changed the chemical characteristic of this amino acid residue from without a side chain (glycine) to one with a large side chain containing negative charge (glutamate) that could interfere with substrate binding and may affect folding the protein. There are no other variations at this position among the 71 resequenced sorghum lines in Phytozome v.12 [33,34]. This allele is present in all reads aligning to this position in the *bmr19* bulk and absent in all reads of the normal bulk, which all have a G at this position.

### 3.5. Association of the bmr19-ref Allele with the Brown Midrib Phenotype Outside the Mapping Population

**Figure 4:**
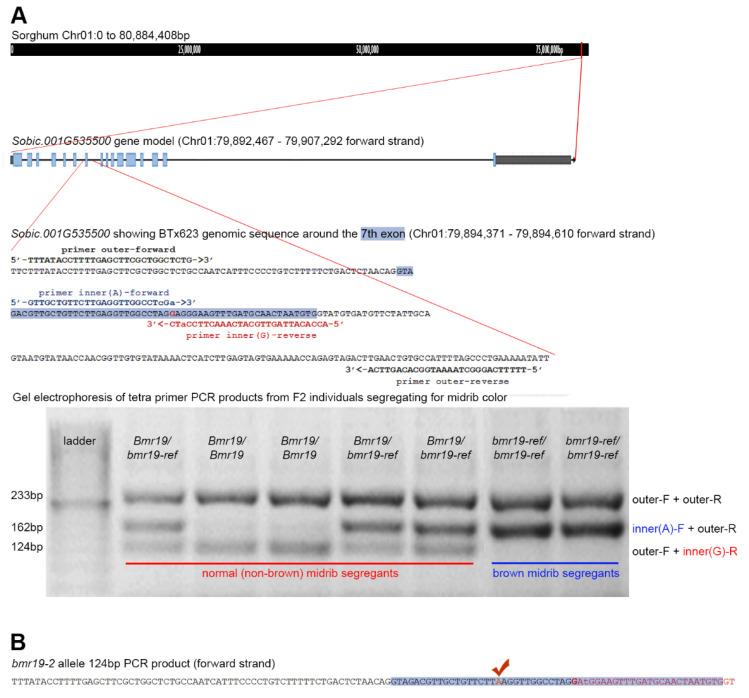
Detection of alleles at *Bmr19* using DNA based markers. (**A**) Location and scheme for the primer set used to detect the SNP distinguishing the *bmr19-ref* (A) from wildtype *Bmr19* (G) alleles. Based on the method described by Medrano and de Oliveira (2014) [37], four primers in the PCR amplify three possible products. The outer primers (outer-F + outer-R) result in a control amplicon of 233 bp in all samples. Mismatches are introduced near the 3′ ends of both inner primers (small case) which both end on the SNP. In the PCR with a wildtype template allele (*Bmr19*), only the inner(G)-R primer works to give a second amplicon of 124 bp with outer-F since the second mismatch at the 3′ end of inner(A)-F is not tolerated. Only when there is an “A” at this position, as occurs in *bmr19-ref*, will the inner(A)-F give an amplicon with outer-R of 162 bp. Because of the double mismatch at the 3′ end, inner(G)-R does not work with the mutant template. The PCR products are separated by size with gel electrophoresis. In F_2_ populations derived from bmr19 crossed with wildtype that segregate for midrib phenotypes, all brown midrib individuals give two bands (233 + 162 bp) corresponding to the *bmr19-ref*/*bmr19-ref* genotype. Normal plants give either two bands (233 + 124 bp) if they are homozygous (*Bmr19*/*Bmr19*) or three bands (233 + 162 + 124 bp) if they are heterozygous (*Bmr19*/*bmr19-ref*). (**B**) Due to the proximity of the SNP distinguishing the *bmr19-2* allele, the same tetra primer set was useful for confirming F_1_s from the allelism test cross of emasculated bmr19 with 2354. PCR products from true F_1_s (*bmr19-ref*/*bmr19-2*) give three bands like the heterozygotes pictured above while any selfs due to incomplete emasculation (*bmr19-ref*/*bmr19-ref*) give only two bands. Sequencing of the smallest band (124 bp) from F_1_ plants showed the *bmr19-2* mutation G → A at the position indicated in orange. The genotype *bmr19-ref*/*bmr19-2* had a brown midrib phenotype, confirming that the mutations are allelic.

An allele specific primer set was designed to convert the SNP to a size polymorphism upon PCR amplification and gel electrophoresis (Figure 4). The *bmr19-ref* allele (A) was resolved from the wildtype allele (G) by 38 bp difference in product size. This marker was run against the nine lines constituting the normal bulk, which all showed G and the nine members of the *bmr19* bulk which all gave amplicons corresponding to A at this position. The marker was also used on the original brown midrib mutant lines of Porter et al. [13] and these all, with the exception of bmr19 which had the A allele, gave bands corresponding to the wildtype allele. We also checked two F_2_ populations derived from the original bmr19 line, one from a cross with BTx623 and the second with IS4225, both with non-brown phenotypes. The marker representing the A allele always cosegregated with the visually scored brown midrib plants and the normal (non-brown midrib) segregants showed either the G allele or both a G and A allele, representing heterozygotes (Figure 4). The ratios of brown midrib to normal plants in the (BTx623 × bmr19)F_2_ fit a 3:1 ratio ($x^{2}$ = 0.02; *p* = 0.889), with 35 progeny having the genotype *bmr19-ref*/*bmr19-ref*, 69 with *Bmr19*/*bmr19-ref* and 45 with *Bmr19*/*Bmr19*. In the (bmr19 × IS4225)F_2_ population, the number of brown midrib individuals also fit a 3:1 ratio ($x^{2}$ = 0.095; *p* = 0.758), all the brown midrib mutants (69 plants) were homozygous for the A allele (*bmr19-ref*/*bmr19-ref*). This F_2_ population had 143 heterozygous individuals and 73 were homozygous for the wildtype allele (GG) consistent with the hypothesis that the bmr19 mutant phenotype is caused by a recessive mutation in a single locus. From these observations, G → A transition mutation in the *bmr19-ref* allele of *Sobic.001G535500* on the forward strand at position Chr01:79,894,470 likely results in the brown midrib phenotype of the original bmr19 donor.

### 3.6. Alternate Alleles

One of the four variants at this locus, which we named *bmr19-2*, also resulted in a brown midrib phenotype (Table 2). This line (2354) contains a G → A transition mutation at Chr01:79,894,457 resulting in the amino acid substitution E236K. Like *bmr19-ref*, this substitution occurs in the Mur-ligase domain. Here, the negatively charged glutamate is changed to a positively charged lysine residue, perhaps also interfering with substrate binding. The three other EMS derived lines from BTx623 contained missense mutations with substitutions of similar amino acids, even though these substitutions also occur in the Mur ligase domain. Line 0623 has a C → T substitution relative to the reference genome at Chr01:79,894,180 resulting in a predicted A221V substitution whereby a hydrophobic alanine residue is replaced with valine, also hydrophobic. In line 2465, a G → A at Chr01:79,895,561 changes the predicted 378th codon from one for serine, a polar residue, to one for asparagine which is also polar. Line 0662 has two variations in its annotated coding regions with respect to the reference. The first is a G → A at Chr01:79,894,460 causing a valine (nonpolar) to isoleucine (also nonpolar) amino acid substitution at the 237th residue of the predicted protein. The second is a synonymous G → A mutation at Chr01:79,896,564 at the 550th codon for an arginine residue. None of the variations at *Sobic.001G535500* carried by EMS lines 0623, 2465 and 0662 result in brown midrib phenotypes. An additional natural variant allele was revealed in the sequence of sorghum lines Ajabsido (PI 656015; SRR2759170), Feterita Gishesh (PI 152651; SRR2759175), Koro-Kollo (PI 656065; SRR2759174) and Tx436 (PI 561071; SRR2759165). These share the common variation from the reference genome of C → A at Chr01:79,893,872 resulting in a serine (polar) to tyrosine (also polar) amino acid substitution at the 185th residue. Like the three innocuous EMS mutations, these variants do not have a brown midrib phenotype. The *bmr19* bulk also shares this variation from the reference, but given the presence of this mutation in the normal bulk and by inference among the majority of the recurrent parents of the introgression lines, we ruled it out as the cause of the brown midrib phenotype.

The allelic nature of the causal mutations was confirmed by crossing 2354 onto the original bmr19 line. Since the female (*bmr19*) had to be emasculated to conduct the cross, the progeny was checked for self-pollination events resulting from incomplete emasculation with the same tetra primers used to track the *bmr19-ref* allele in the F_2_ populations described above and in Figure 4. All of the progeny showed the brown midrib phenotype. All the progenies were true F_1_s (*bmr19-ref*/*bmr19-2*) and these were distinguishable from any progeny resulting from self-pollination by the presence of a third PCR product of 124 bp. Confirmation of the *bmr19-2* allele in the heterozygotes was achieved by sequencing this PCR product which showed an “A” at the position corresponding to Chr01:79,894,457 of the genomic template. The identification of a second mutant allele, *bmr19-2*, and the brown midrib phenotype associated with it in the homozygous state or in heterozygous combination with the *bmr19-ref* allele confirms the identity of *Bmr19* as *Sobic.001G535500*.

### 3.7. Effects on Feedstock

**Figure 5:**
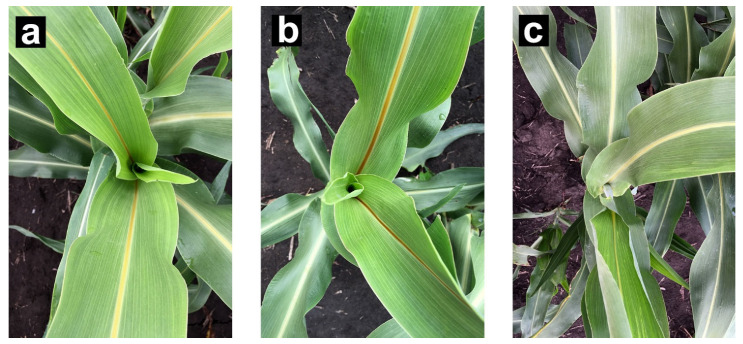
Visible midrib phenotypes of *bmr19* mutants and wildtype in the BTx623 background. Photos taken from field grown plants at early boot stage: (**a**) brown midrib line (BTx623ms3 × bmr19)BC_2_F_4_, carrying the *bmr19-ref* allele; (**b**) brown midrib BTx623 EMS line 2354, carrying the *bmr19-2* allele; (**c**) normal midrib BTx623, carrying the wildtype (*Bmr19*) allele.

In order to determine the effects of the *bmr19* mutations on lignocellulosic quality of sorghum feedstock, we compared stover of the introgression line carrying the *bmr19-ref* allele [(BTx623ms3 × bmr19)BC_2_F_4_] with BTx623 and EMS line 2354 (BTx623 derivative carrying the *bmr19-2* allele). Both mutant lines were slightly later in maturity than BTx623, but similar in plant architecture, distinguished mainly by the color of their midribs (Figure 5). The stover for fiber analysis was collected from all lines on the same date when all plants had reached maturity. Similar to earlier compositional analysis of the original bmr19 line [14], we found slight but significant reductions with respect to wildtype in lignin content of biomass in both BTx623 derivatives bearing *bmr19* mutations (Table 3 and Table 4). These reductions are less severe than in BTx623 derivatives bearing *bmr2*, *bmr6* or *bmr12* mutations (Table 3). In terms of the lignin subunit yields from thioacidolysis, both *bmr19* mutations cause a slight but significant increase in H-subunits, although these remain relatively minor constituents of lignin polymers in wildtype and mutant lines. Both G- and S-subunits are reduced in *bmr19* mutants relative to wildtype. Reduction of the single methoxylated G-subunits, ranging from 16–25%, are approximately twice the reductions (7–14%) of the double methoxylated S-subunits, reflected in the lower G/S ratio of these mutants with respect to wildtype (Table 5). The changes in monomer content appear to be slightly more pronounced in the line bearing the *bmr19-ref* allele, particularly in the reduction of G-subunits.

## 4. Discussion

### 4.1. Power of the Analysis of Whole Genome Resequenced Bulk Comparisons

Whole genome resequencing of bulked DNA from near inbred lines (most at F_4_ or F_5_) carrying particular brown midrib phenotypes compared to bulked DNA from wildtype parents in the original crosses successfully distinguished the causal mutant and wildtype alleles for these phenotypes when the donor mutant alleles were shared among the members of the *bmr* bulks. This occurred for the *bmr2-ref* allele and *bmr6-ref* allele, despite the “contamination” of these mutant bulks with alternate alleles. Mixed alleles undermined detection of a candidate SNP-index peaks by the analytical protocol employed in the *bmr12* bulk because the donor mutant alleles were more evenly mixed with five of the nine members of the *bmr12* bulk having the *bmr12-18* allele, three with the *bmr12-ref* allele and one with the *bmr12-7* allele (Table 1). Although the analysis failed to pick a common polymorphism between the *bmr12* and normal bulks in the aligned consensus sequence, the presence of all these mutant alleles among the reads aligning to this locus can be seen using IGV software [35] in the *bmr12* bulk. The analysis was further hindered by polymorphisms among the members of the normal bulk of functionally inert indels in the *Bmr12* gene. Recurrent parents Sudan Zera-Zera and P9401 share these polymorphisms (missing *Olo24* and two repeat units in the SSR) with the bmr7, bmr12 and bmr18 donor parents. Because the analysis filtered the consensus assembly by comparison to Sudan Zera-Zera, these polymorphisms skewed the results in this region.

### 4.2. The Bmr19 Locus

The analysis showed a genomic interval with a SNP-index peak between the *bmr19* and normal bulks and within this candidate genomic interval at *Sobic.001G535500*, a single SNP that is strongly suggested to be the causal mutation. This *bmr19-ref* allele represents a missense mutation G240E occurring within the annotated Mur-ligase domain associated with binding the glutamic acid substrate. All brown midrib introgression lines derived from the original bmr19 line contain this variation and the mutation always cosegregated with the brown midrib phenotype in F_2_ populations. An alternate allele at this locus confirmed the identity of *Sobic.001G535500* as *Bmr19*. The E236K mutation in the brown midrib EMS line 2354 carrying the *bmr19-2* allele (Table 2) similarly affects a residue charge in the Mur ligase domain of the gene product. Less severe amino acid substitutions in which the charge characteristics of the residues remain unchanged in this domain did not cause a mutant phenotype.

The predicted product of *Sobic.001G535500* or *Bmr19* is a FPGS, an enzyme involved in folate (C1) metabolism. Its link to lignin biosynthesis is best explained in two publications describing the maize *bm4* mutant [41] and the *fpgs1* mutant of *Arabidopsis* [43]. Briefly, the tie of C1 metabolism to lignin is that it synthesizes the methyl donor, SAM, that COMT and CCoAOMT use to form the methoxy groups of coniferyl and sinapyl alcohols which ultimately become the G and S subunits of the lignin polymer. The actual role of FPGS in the biosynthesis of SAM is to add glutamic acid to tetrahydrofolate (THF) to form a short polyglutamate tail important for efficient and properly compartmentalized function of THF [44] in its role as cofactor in the downstream methylation of homocysteine to methionine, a direct precursor of SAM. Both polyglutamylated folates and SAM are vital in the biosynthesis of several primary and secondary plant metabolites, including purines, the amino acids methionine and serine and betaines [45], so a complete loss of FPGS activity would likely be lethal. A second predicted FPGS (*Sobic.001G201900*) is present in the sorghum genome, which may be sufficiently redundant to prevent these deleterious effects. One might expect that the mutations in *bmr19-ref* and *bmr19-2* impair lignin biosynthesis by limiting the supply of the cofactor SAM to the O-methyltransferases in the pathway thereby impairing the synthesis of coniferyl and sinapyl monolignols. Our analysis of lignin composition via thioacidolysis in *bmr19* stover showed that S-subunits were less reduced than G-subunits with respect to wildtype. A decreased G:S ratio of lignins was also reported in maize *bm4* mutant stalks [41,42,46]. This suggests that the C1 metabolites feeding the guaiacyl branch of the lignin biosynthetic pathway are disproportionately limited. In *Arabidopsis*, expression of other genes in the lignin biosynthetic pathway was reportedly downregulated in *fpgs1* mutants, implying some feedback control that rations the limited C1 metabolites, including SAM, to cellular functions more vital than lignification [43]. The introgression lines carrying the *bmr19-ref* mutation will be excellent materials in which to monitor any possible regulatory effects on other lignin biosynthetic genes in sorghum.

Considering the relatively minor effects of *bmr19* alleles on lignocellulosic properties of sorghum with respect to *bmr6* and *bmr12*, these mutants at first seem not to be as useful as the other *bmr* mutations to improving saccharification of feedstock for biofuel production. Future work may generate stronger *bmr19* alleles that more severely impair or eliminate this specific FPGS product feeding sorghum lignin biosynthesis with possible improvements to feedstock quality.

A FPGS in switchgrass (*Panicum virgatum*) encoded by *Pavir.Ib00114* (*PvFPGS1*) with high homology (93.8% identical + positively scoring residues in alignment according to Phytozome v.12) to sorghum *Bmr19* was targeted by RNAi to either knock-down or knock-out the activity of one of three FPGS in transgenic plants evaluated in the field for harvestable biomass and ethanol yield [47]. Transgenic lines in which *PvFPGS1* expression was essentially knocked out had severe consequences on plant biomass yield. Similar to the current analyses of *bmr19* sorghum mutants, lignin content and composition of moderately knocked-down *PvFPGS1* transgenic switchgrass was little changed from controls. However, in certain of these transgenic switchgrass lines ethanol yield was improved by as much as 18%. This was despite no obvious improvement in sugar release efficiency in these transgenics relative to control [47]. The authors of the switchgrass study monitored global gene expression in their transgenic lines through RNA-seq and speculate that the increased ethanol yield in their low-to-moderate *PvFPGS1* knock-down transgenics may be due to perturbations in gene expression through metabolite feedback that alter the expression of genes whose products ultimately affect the level of lignin polymerization in cell walls rather than the actual production of monolignols [47].

Having identified the *Bmr19* locus as encoding an enzyme peripheral to the canonical monolignol biosynthetic pathway, the metabolic targets for engineering improved lignocellulosic feedstock from sorghum has expanded. Improvements to ethanol yield from sorghum bearing the *bmr19* mutations described herein may not be great but other alleles at this locus that more severely affect FPGS function may be more useful. Plants with both the *fpgs1* and *ccoaomt1* mutations in *Arabidopsis* increased the enzymatic polysaccharide hydrolysis efficiencies over those of single mutants by up to 20% without adverse growth effects [48]. Similar double mutants like *bmr12bmr19* bearing sorghum could result in better ethanol yields.

## Figures and Tables

**Table 1:** Constitutions of the resequenced bulks and pedigrees of brown midrib introgression lines.

Normal (Non-Brown) Bulk		*bmr2* Bulk		*bmr6* Bulk		*bmr12* Bulk		*bmr19* Bulk	
Pedigree	Relevant Alleles in Homozygous State	Pedigree	Relevant Alleles	Pedigree	Relevant Alleles	Pedigree	Relevant Alleles	Pedigree	Relevant Alleles
PP290	*Bmr2*, *Bmr6*, *Bmr12*, *Bmr19*	(bmr2ms3 × PP290)BC_3_F_5_	*bmr2-ref*	(bmr6ms3 × PP290)BC_3_F_5_	*bmr6-ref*	(bmr18ms3 × PP290)BC_3_F_5_	*bmr12-18*	(bmr19ms3 × PP290)BC_2_F_5_	*bmr19-ref*
PRL983999	*Bmr2*, *Bmr6*, *Bmr12*, *Bmr19*	(bmr2ms3 × PRL983999)BC_3_F_4_	*bmr2-ref*	(bmr6ms3 × PRL983999)BC_3_F_4_	*bmr6-ref*	(bmr12ms3 × PRL983999)F_4_	*bmr12-ref*	(bmr19ms3 × PRL983999)F_4_	*bmr19-ref*
Sudan Zera-Zera	*Bmr2*, *Bmr6*, *Bmr12*, *Bmr19*	(bmr2ms3 × Sudan Zera-Zera)F_4_	*bmr2-ref*	(bmr6ms3 × Sudan Zera-Zera)BC_3_F_4_	*bmr6-ref*	(bmr18ms3 × Sudan Zera-Zera)BC_3_F_4_	*bmr12-18*	(bmr19ms3 × Sudan Zera-Zera)BC_2_F_5_	*bmr19-ref*
PU216B	*Bmr2*, *Bmr6*, *Bmr12*, *Bmr19*	(bmr2ms3 × PU216B)BC_2_F_5_	*bmr2-ref*	(bmr6ms3 × PU216B)BC_3_F_5_	*bmr6-ref*	(bmr12ms3 × PU216B)BC_3_F_5_	*bmr12-ref*	(bmr19ms3 × PU216B)BC3F3	*bmr19-ref*
P942242B	*Bmr2*, *Bmr6*, *Bmr12*, *Bmr19*	(bmr2ms3 × P942242B)BC_3_F_4_	*bmr2-ref*	(bmr6ms3 × P942242B)BC_3_F_4_	*bmr6-ref*	(bmr18ms3 × P942242B)BC_3_F_4_	*bmr12-18*	(bmr19ms3 × P942242B)BC_2_F_4_	*bmr19-ref*
P90344	*Bmr2*, *Bmr6*, *Bmr12*, *Bmr19*			(bmr6ms3 × P90344)BC_2_F_5_	*bmr6-ref*	(bmr18ms3 × P90344)BC_2_F_5_	*bmr12-18*	(bmr19ms3 × P90344)BC_3_F_3_	*bmr19-ref*
P90812	*Bmr2*, *Bmr6*, *Bmr12*, *Bmr19*	(bmr2ms3 × P90812)F_4_	*bmr2-ref*	(bmr3ms3 × P90812)BC_2_F_5_	*bmr6-3*	(bmr12ms3 × P90812)BC_2_F_5_	*bmr12-ref*	(bmr19ms3 × P90812)BC_2_F_5_	*bmr19-ref*
Tx623B	*Bmr2*, *Bmr6*, *Bmr12*, *Bmr19*	(bmr2ms3 × Tx623B)BC_3_F_5_	*bmr2-ref*	(bmr6ms3 × Tx623B)BC_3_F_5_	*bmr6-ref*	(BTx623ms3 × bmr18)BC_2_F_4_	*bmr12-18*	(BTx623ms3 × bmr19)BC_2_F_4_	*bmr19-ref*
P9401	*Bmr2*, *Bmr6*, *Bmr12*, *Bmr19*	(bmr5ms3 × P9401)BC_4_F_5_	*bmr2-5*	(bmr3ms3 × P9401)BC_4_F_5_	*bmr6-3*	(bmr7ms3 × P9401)BC_4_F_5_	*bmr12-7*	(P9401ms3 × bmr19)BC_3_F_5_	*bmr19-ref*

**Table 2:** Lines derived from ethyl methanesulfonate mutagenesis with variation in *Sobic.001G535500* with respect to the reference genome.

Line Name	NPGS Accession Number	SRA Accession Number	Mutation Position on Chr01 *	Base Pair Change	Amino Acid Change Chemical Characteristic Change	Leaf Midrib Phenotype	Mutant Allele Name
**0623**	PI 677943	SRR2759749	79,894,180	C → T	A221V hydrophobic → hydrophobic	normal	
**2354**	PI 678119	SRR2759203	79,894,457	G → A	E236K negative → positive	brown	*bmr19-2*
**2465**	PI 678132	SRR2759494	79,895,561	G → A	S378N polar → polar	normal	
**0662**	PI 677955	SRR2759755	79,894,460	G → A	V237I nonpolar → nonpolar	normal	
**bmr19**			79,894,470	G → A	G240E nonpolar → negative	brown	*bmr19-ref*

* position according to Phytozome *Sorghum bicolor* v.3.1.1.

**Table 3:** Fiber analysis of greenhouse grown wildtype and *bmr* introgression lines in the BTx623 background.

Line	Relevant Alleles in Homozygosity	Neutral Detergent Fiber (Cellulose, Hemicellulose and Lignin)		Acid Detergent Fiber (Cellulose and Lignin)		Acid Detergent Lignin (Lignin)	
		Mean *	Std Dev	Mean	Std Dev	Mean	Std Dev
BTx623	*Bmr2*, *Bmr6*, *Bmr12*, *Bmr19*	63.1 *a*	5.7	37.5 *a*	4.0	5.0 *a*	0.6
(bmr2ms3 × Tx623B)BC_3_F_5_	*bmr2-ref*	58.9 *ab*	6.0	34.1 *ab*	3.9	2.6 *c*	0.4
(bmr6ms3 × Tx623B)BC_3_F_5_	*bmr6-ref*	55.8 *b*	8.1	31.8 *b*	5.4	2.5 *c*	0.3
(BTx623ms3 × bmr18)BC_2_F_4_	*bmr12-18*	57.6 *b*	4.2	32.6 *b*	2.7	2.5 *c*	0.3
(BTx623ms3 × bmr19)BC_2_F_4_	*bmr19-ref*	59.7 *ab*	5.6	33.5 *ab*	4.3	3.6 *b*	0.2
BTx623 EMS line 2354	*bmr19-2*	62.2 *ab*	3.5	34.8 *ab*	2.5	3.7 *b*	0.9

* Means with the same letter are not significantly different. Values expressed as g/kg dry weight.

**Table 4:** Fiber analysis of field grown mutant (*bmr19*) and wild type (*Bmr19*) sorghum lines in the BTx623 background.

Genotype *	Neutral Detergent Fiber (Cellulose, Hemicellulose and Lignin)		Acid Detergent Fiber (Cellulose and Lignin)		Acid Detergent Lignin (Lignin)	
	Mean †	Std Dev	Mean	Std Dev	Mean	Std Dev
*Bmr19*	55.8 *b*	1.7	29.1 *b*	1.0	4.6 *a*	0.2
*bmr19-ref*	56.1 *b*	2.2	28.2 *b*	1.2	3.9 *c*	0.2
*bmr19-2*	61.9 *a*	1.8	31.8 *a*	1.5	4.3 *b*	0.2

* *Bmr19* = wildtype allele in homozygosity in BTx623; *bmr19-ref* = mutant allele in homozygosity in (BTx623ms3 × bmr19)BC_2_F_4_; *bmr19-2* = mutant allele in homozygosity in line 2354. † Means with the same letter are not significantly different. Values expressed as g/kg dry weight.

**Table 5:** Lignin monomer [*p*-hydroxyphenyl (H), syringyl (S), guaiacyl (G)] yield by thioacidolysis of field grown biomass from BTx623 and derivatives.

Genotye *	H (µg/g Dry Weight)		G (µg/g Dry Weight)		S (µg/g Dry Weight)		G/S	
	Mean †	Std Dev	Mean	Std Dev	Mean	Std Dev	Mean	Std Dev
*Bmr19*	6607.1 *a*	852.3	169,109.5 *a*	25,095.0	104,810.2 *a*	21,144.7	1.6 *a*	0.1
*bmr19-ref*	7629.6 *b*	1134.5	126,173.5 *c*	17,264.5	89,806.9 *b*	16,331.9	1.4 *b*	0.1
*bmr19-2*	7239.5 *ab*	951.1	141,872.7 *b*	15,806.5	97,702.2 *ab*	12,935.2	1.5 *b*	0.1

* *Bmr19* = wildtype allele in homozygosity in BTx623; *bmr19-ref* = mutant allele in homozygosity in (BTx623ms3 × bmr19)BC_2_F_4_; *bmr19-2* = mutant allele in homozygosity in line 2354. † Means with the same letter are not significantly different.

## Data Availability

The data presented in this study, are contained within the article.
